# Characterization of Malignant Melanoma Using Vibrational Spectroscopy

**DOI:** 10.1100/tsw.2005.1

**Published:** 2005-03-18

**Authors:** Ziad Hammody, Ranjit Kumar Sahu, Shaul Mordechai, Emanuela Cagnano, Shmuel Argov

**Affiliations:** ^1^ Department of Physics, Ben Gurion University of the Negev, Beer-Sheva, Israel; ^2^ Department of Pathology, Soroka University Medical Center, Ben Gurion University of the Negev, Beer-Sheva, Israel

**Keywords:** FTIR-MSP, melanoma, nevus, peak shifts, cancer, Israel

## Abstract

Malignant melanoma, a malignant neoplasm of epidermal melanocytes is the third most common skin cancer. In many cases, melanoma develops from nevus, which is considered as the nonmalignant stage. Fourier transform infrared microspectroscopy (FTIR-MSP), which is based on characteristic molecular vibrational spectra of cells, was used to investigate spectral differences between melanoma, nevus, and the corresponding normal epidermis. In the present work, FTIR-MSP was performed on formalin-fixed biopsies of melanoma and nevi along with the adjoining histologically normal epidermis to understand the biochemical variations from the epidermis and identify suitable parameters for differentiation of nevi from melanoma. The comparative analysis of various parameters calculated from the spectral data of the normal epidermis and the abnormal regions showed that the changes in the nucleic acids was a significant indicator of the abnormal nature of the tissues. The RNA/DNA ratio was decreased in case of both melanoma and nevus compared to the epidermis. The amide II/amide I ratio was greater for nevus and melanoma compared to the epidermis. In contrast to other organs, the analysis of carbohydrates was not found as a suitable indicator in case of malignant melanoma. Shifts in band wave number were found to be a major distinguishing feature between the melanoma and compound nevi. The present study helps in the identification of spectral features suitable for distinction of melanoma from nevus that appear similar even in FTIR spectral features and thus can pave the way for development of *in vivo* screening systems based on these diagnostic markers.

## INTRODUCTION

Skin cancer is the most common cancer in the U.S., affecting about 1,000,000 Americans every year. Melanoma accounts for about 4% of skin cancer cases, but it causes about 79% of skin cancer deaths. The American Cancer Society[1] estimates that in 2004 there will be 55,100 new cases of melanoma in this country. About 7,910 people will die of this disease. The number of new cases of melanoma in the U.S. is on the rise. Thus, this cancer is highly important from the point of pathological diagnoses. High risk factors for melanoma include presence of pigmented lesions (dysplastic or atypical nevus), with several large nondysplastic nevi, with many small nevi, with moderate freckling, or with familial dysplastic nevus syndrome[2,3]. Both malignant melanoma and compound nevi are found on the skin surfaces so that surface sensing techniques that are noninvasive like Fourier transform infrared (FTIR) coupled with fiber optics evanescent spectroscopy (FEWS) systems can be developed for *in vivo* distinguishing between the two groups.

Distinction between normal and malignant tissues and cells[4,5] using FTIR spectroscopy has been an area of growing interest to both pathologists and the biomedical industry due to the rapid data acquisition possibility and specificity of normal and abnormal tissue signatures in the FTIR spectra. In the present study, a comparison was made between the biochemical changes occurring in compound nevi and melanoma with respect to the adjoining normal epidermis, which was used as the reference using paraffin-embedded biopsies. Earlier works on human basal cell carcinoma by FTIR spectroscopy[6,7,8] have not dealt exclusively with FTIR-MSP of formalin-fixed biopsies. Thus, it became important to study the validity of FTIR-MSP in diagnosis of melanoma from formalin-fixed biopsies commonly used by pathologists, since a large number of samples are available for standardization of the techniques and searching of diagnostic features for detection of malignancy in tissues, due to their different chemical composition. Moreover, the similarities between the two forms of skin abnormality have not been dealt with, which is also important during diagnosis to decrease the false negative.

In the present work, we studied the FTIR-MSP and histopathology in tandem on consecutive sections of formalin-fixed biopsies. The work highlights the commonalities between two pathological conditions: compound nevi and melanoma in comparison to the appropriate normal tissues. The comparison between the epidermis and benign/malignant tissues from the same patients using FTIR-MSP will reduce heterogeneity arising when comparing between different patients and highlight the similarities between the two types of pathological conditions at the biochemical level. Such information can be used to create model variations in tissue imaging which can be used to diagnose the biopsies *ex vivo* or used later for *in vivo* data correlations.

## METHODS

Formalin-fixed, paraffin-embedded tissue from patients with melanoma and patients having nevi were retrieved with their consent from the histopathology files of Soroka University Medical Center (SUMC), Beer-Sheva, Israel. The tissues from benign compound nevi and malignant melanoma (superficial spreading type) were taken from different regions of the skin. Two adjacent paraffin sections were cut from each biopsy. One was placed on zinc-selenium slide and the other on glass slide. This procedure was carefully followed to assure that the tissue sections were practically identical[9]. The thickness of the tissue samples was ~10 μm**.** The first slide was deparaffinized using xylol and alcohol and used for FTIR measurements. The second slide was stained with hematoxylin and eosin (HE) for parallel, on-line histology review. Microscopic FTIR measurements in transmission mode were performed using the FTIR microscope IRscope II with a mercury-cadmium-telluride (MCT) detector, coupled to the FTIR spectrometer (Bruker Equinox model 55/S, OPUS software). An expert pathologist examined the tissue histology on-line to assure accurate determination of the measured microscopic sites. For each biopsy, at least five measurements at different locations were made and the average spectra analyzed. The data were normalized and analyzed using the OPUS software. All the spectra were normalized with respect to amide II (~1546 $cm^{-1}$)[10] for the region 600–1600 $cm^{-1}$ and the $CH_2$ antisymmetric peak (~2920)[10] for the region 2800–3000 $cm^{-1}$.

## RESULTS

Representative spectra in the Mid-IR region of normal epidermis, compound nevus, and melanoma from biopsies are displayed in Fig. 1. In case of nevi and melanoma, the histology was compared with respect to the normal epidermis from the peripheral regions of the biopsy as shown by histology in the HE-stained slides. Thus, the nevi and melanoma were compared after standardization to the corresponding epidermis from the same patient to avoid any heterogeneity, which naturally occurs between different patients. It was seen that the normal epidermis had a lower absorbance in the region 900–1185 $cm^{-1}$ compared to the compound nevus as well as melanoma. In case of melanoma, the absorbance was slightly higher compared to the epidermis in some regions (800–1200 $cm^{-1}$) corresponding mainly to phosphates and carbohydrates and also in other regions corresponding to absorbance due to lipids, nucleic acids, and proteins (1200–1600 $cm^{-1}$). The expanded spectra in the range between 2800–3000 $cm^{-1}$ are displayed in Fig. 1b after baseline correction. It is seen that in both types of abnormality, there is a decrease in the absorbance in the antisymmetric $CH_3$ region and increase in the $CH_2$ symmetric region when compared to the normal epidermis. Thus, it became important to quantify the spectral changes to identify parameters for distinction of these two abnormalities.

The amount of glucose (carbohydrates) in the tissues was quantified by the integrated absorbance from the area 1020–1040 $cm^{-1}$. The results are shown in Fig. 2. It was observed that the levels of carbohydrates were higher in case of the melanoma or nevus compared to the epidermis. This could be due to the transfer of carbohydrates to these tissues from the surrounding epidermis leading to a relative increase in the diseased tissues. Moreover, while under normal conditions the cells in the epidermis gradually die and exfoliate, in the case of the melanoma they are in an active phase of growth, thus having carbohydrates for their metabolism. There was a general tendency of this parameter to increase in case of both melanoma and nevus making it an unsuitable parameter to distinguish the nevus and melanoma as seen from Fig. 2. The values were compared using the standard t test and the mean values were not significantly different at 5% indicating that the carbohydrate was unsuitable for utilization in imaging systems. Therefore skin tissues if colored under pseudocoloring programs using this parameter would not differentiate the epidermis from nevus or melanoma and accurately show if the disease was melanoma or nevus. Thus, it was essential to identify other parameters, which provided a better distinction of melanoma and nevus during such image analysis by surface scanning techniques based on IR spectroscopy.

Nucleic acids are important parameters that have been used for distinction of normal and cancerous cells and tissues. The levels of phosphate indicated by the integrated absorbance between 1065–1095 $cm^{-1}$ are presented in Fig. 3(a). It is observed that in both the nevus and epidermis, the levels of phosphate are increased indicating higher phosphate metabolism compared to the surrounding epidermis. It is known that the cell division is altered during both these conditions. Thus, it is imperative that the levels of nucleotides would increase along with nucleic acids like DNA/RNA in these tissues. The amide II/amide I ratios denoting the changes during the melanoma and nevus compared to the epidermis are shown in Fig.3 (b). It is observed that in most cases, the values are higher for the melanoma and the nevi indicating biochemical changes in these tissues compared to the surrounding epidermis. Benedetti et al.[11] had shown that the leukemic cells had a higher ratio compared to the normal cells for this parameter. Since both leukemia and melanoma are considered as malignant and nevus often premalignant, the similar behavior of this parameter in all cases is an indicator of the general trend of biochemical changes during carcinogenesis. The level of DNA compared to RNA is also an indicator of enhanced DNA synthesis and cell division. It is also possible that due to the conformational changes, the DNA signals are increased at a constant level of RNA leading to such variations in the spectra. It is noteworthy that in case of both melanoma as well as nevus, there is a decrease in this parameter which is indicated by the ratio I ($1121cm^{-1}$)/I($1020cm^{-1}$)(Fig. 3c). The variation in this ratio is also observed by considering the RNA/DNA ratio from the antisymmetric phosphate vibrations in the region 1185–1350 where the nucleic acids absorb. Fig. 3d shows such a ratio calculated form the absorbance at 1244 and 1230 $cm^{-1}$ for RNA and DNA, respectively. Thus, there is increased DNA content as evaluated by FTIR-MSP in case of both compound nevus and melanoma compared to the surrounding epidermis. The mean values of the parameters obtained from absorbance of nucleic acids were significantly different when seen by standard t test. However, there was an overlap in the values.

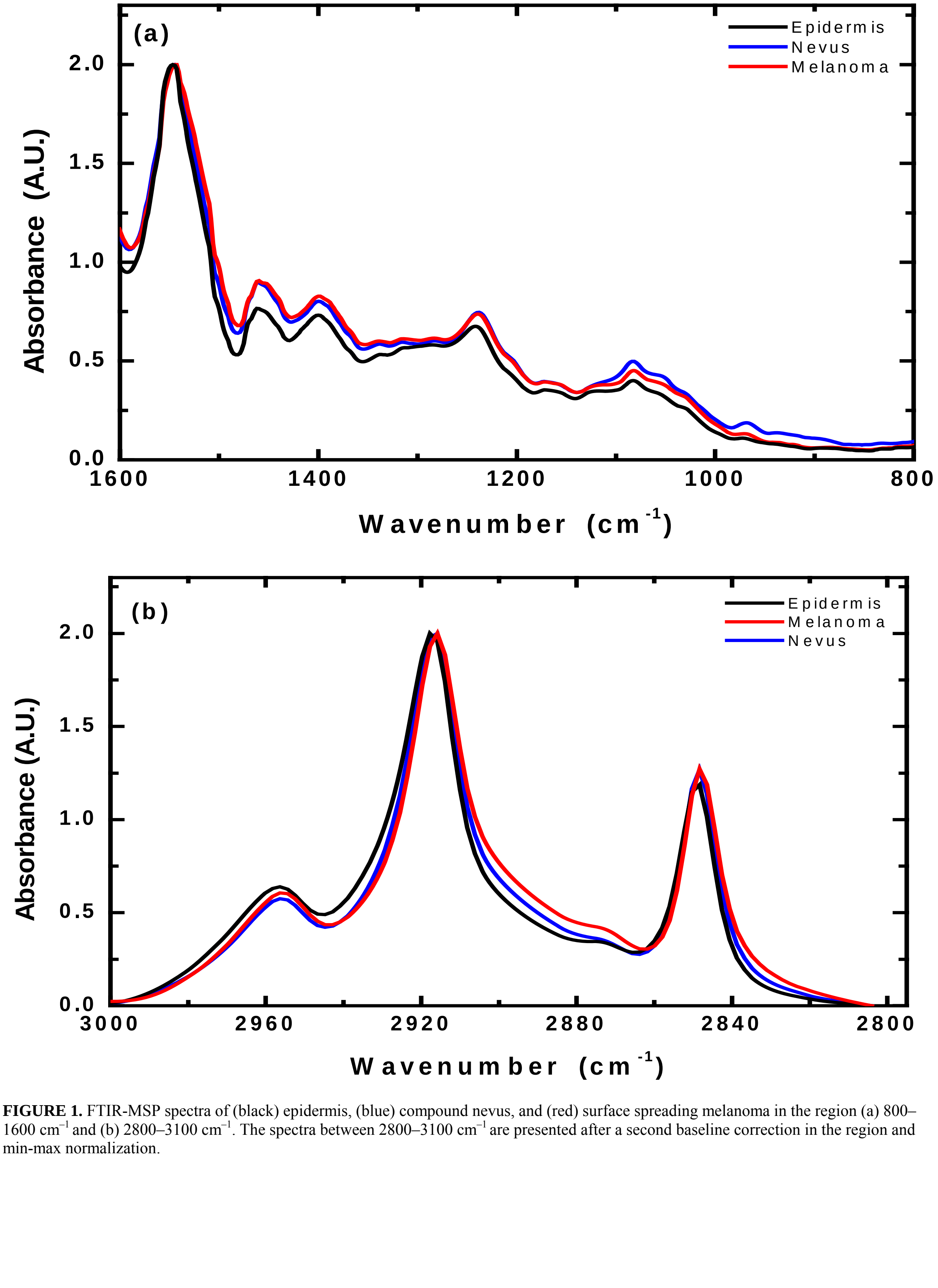

**FIGURE 1.** FTIR-MSP spectra of (black) epidermis, (blue) compound nevus, and (red) surface spreading melanoma in the region (a) 800–1600 $cm^{-1}$ and (b) 2800–3100 $cm^{-1}$. The spectra between 2800–3100 $cm^{-1}$ are presented after a second baseline correction in the region and min-max normalization.

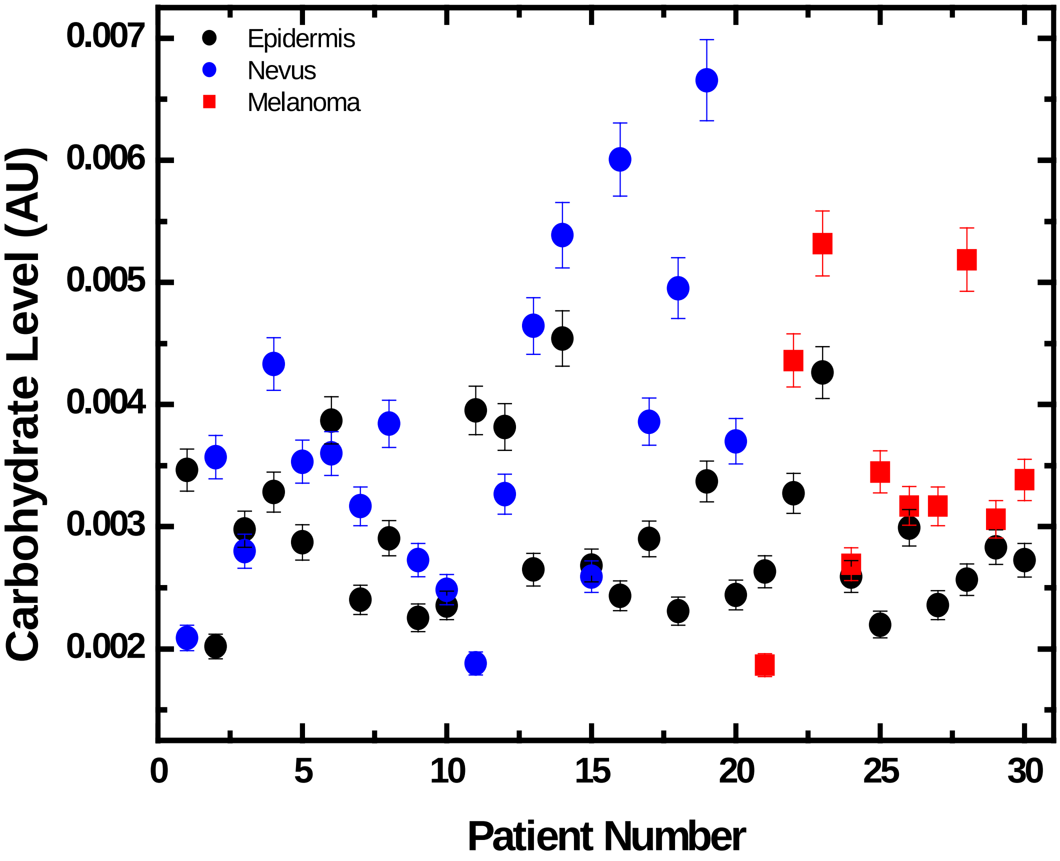

**FIGURE 2.** Variations in the glucose levels between epidermis, compound nevus, and melanoma as quantified from the integrated absorbance between 1020–1040 $cm^{-1}$. The black circles represent the epidermis and the blue circles and red squares represent the nevus and the melanoma, respectively.

It was therefore essential to find additional definitive features to distinguish between the melanoma and nevus for diagnostic purposes. The shift in the peak wave numbers was considered as an additional indicator of changes in the tissue biochemistry. Table 1 indicates some of the relevant peak absorbance and the variations as studied by FTIR-MSP. It is observed that the 1240 $cm^{-1}$ band is shifted toward the lower wave numbers in case of both nevus and melanoma. The difference is more in case of nevus compared to the melanoma when the epidermis is used as a reference. This shift could be due to the increased DNA content as this peak arises due to the balance from both the RNA and DNA and as seen in the figures, the DNA level increase compared to RNA. The presence of shift in both the nevus and melanoma further reiterates this observation.

Similarly, the amide I and amide II peaks are shifted in case of the diseased conditions. The amide II shift occurs towards a lower wave number in case of both nevus and melanoma, but to different degrees. The amide I band shifts toward a lower wave number in the case of melanoma and a higher wave number in the case of nevus. This possibly would be a distinguishing feature of melanoma from nevus. Similar observations are also made regarding the absorbance from the $CH_2$ antisymmetric stretching at ~2920 $cm^{-1}$. In this case however, the shift is towards the lower wave number in the case of nevus and higher wave number in the case of the melanoma.

Thus for identification of melanoma and distinction from nevus, not only the ratios, but also the shifts would play a role. This is especially true as the skin surface (epithelium) does not undergo a progressive change in levels of carbohydrates and phosphates, which can be used as principal components for distinction of normal and malignant tissues[12,13].

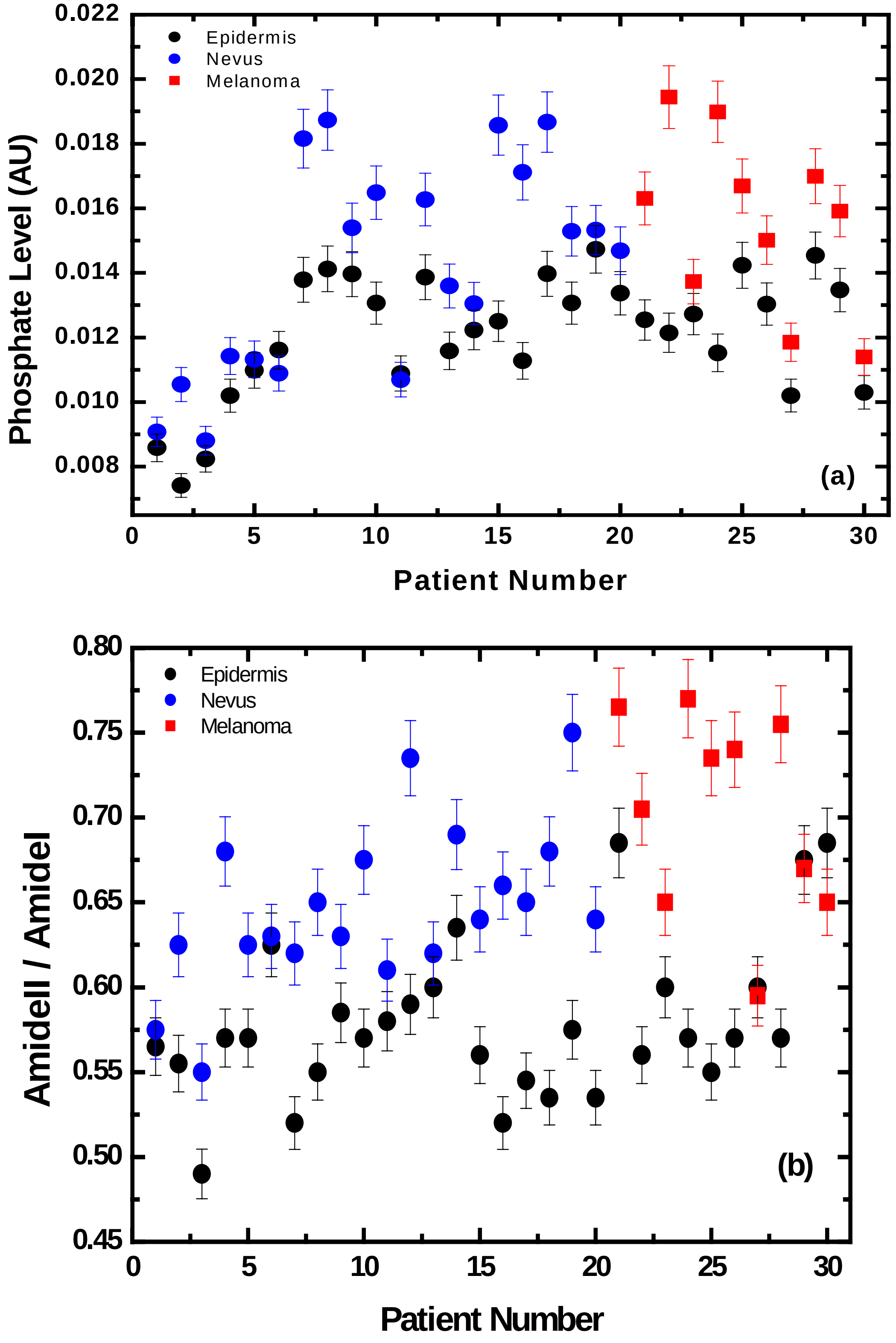

**FIGURE 3.** Variations in the nucleic acid levels between the epidermis and compound nevus/melanoma as quantified from the absorbance (a) integrated area of 1065–1095 $cm^{-1}$, (b) amide I/amide II, (c) I(1121 $cm^{-1}$)/I(1020 $cm^{-1}$), and (d) I(1244 $cm^{-1}$)/I(1230 $cm^{-1}$). The black symbols represent the epidermis and the blue circles and red squares represent the nevus and the melanoma, respectively.

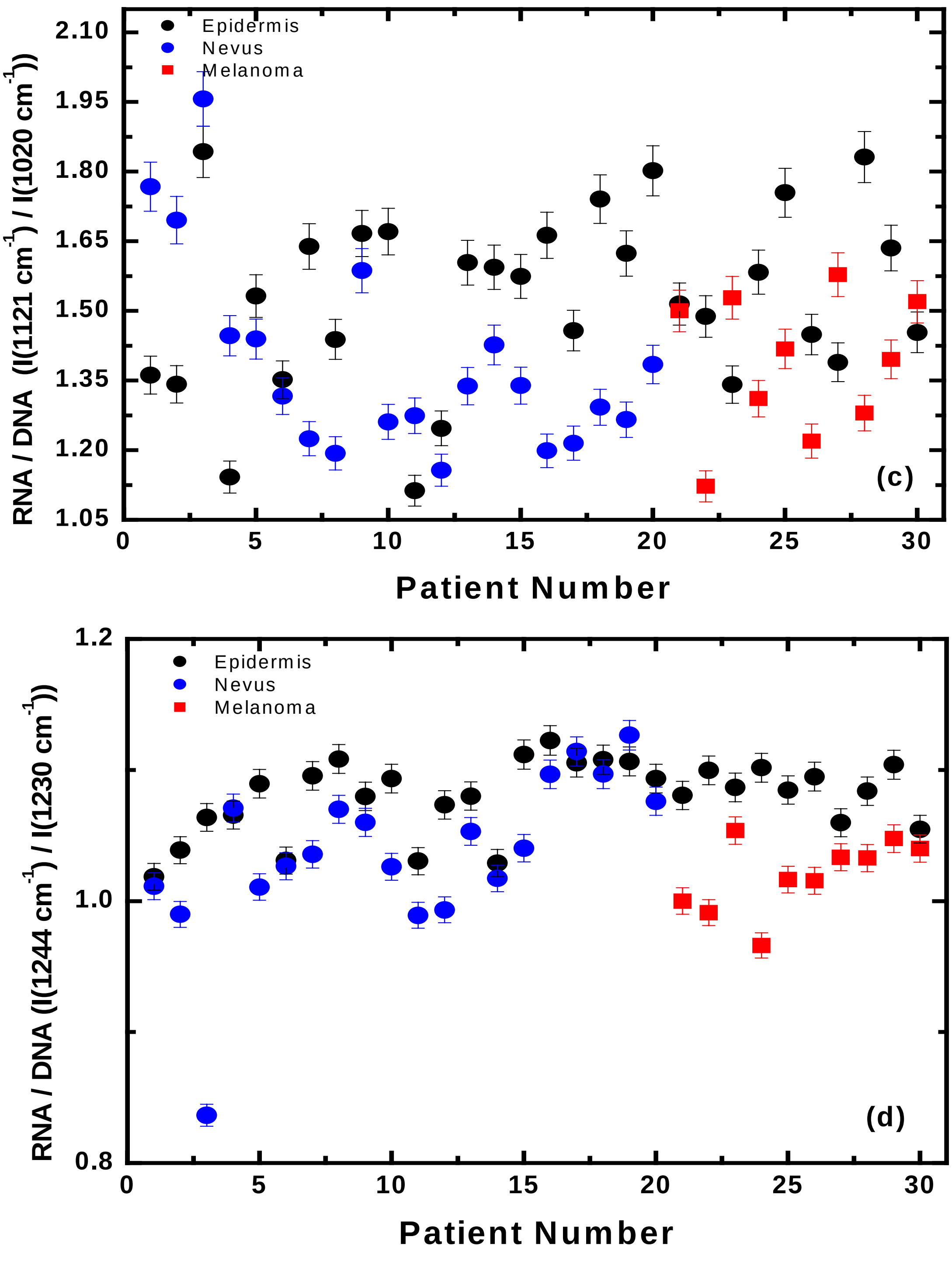

**FIGURES 3c and d**

**TABLE 1**
**Variations in Absorbance Maxima between Epidermis, Nevus, and Melanoma Tissue in the Mid-IR Region as Deduced from Second Derivative Spectra**

| Band | Melanoma | Nevus | Epidermis |
|---|---|---|---|
| DNA (966) | 970.4 ± 0.4 | 970.1 ± 0.3 | 968.5 ± 1.9 |
| $PO_2$ stretching, symmetric (1084) | 1081.2 ± 0.3 | 1082.6 ± 0.2 | 1081.8 ± 0.3 |
| $PO_2$ stretching, antisymmetric (1240) | 1237.9 ± 0.3 | 1235.3 ± 0.4 | 1241.4 ± 0.3 |
| Amide II | 1543.2 ± 0.9 | 1544.6 ± 0.7 | 1545.4 ± 0.4 |
| Amide I | 1648.7 ± 0.7 | 1652.5 ± 0.3 | 1650.9 ± 0.2 |
| $CH_{2,}$stretching, symmetric | 2848.9 ± 0.3 | 2848.3 ± 0.1 | 2849.5 ± 0.2 |
| $CH_{3,}$stretching, symmetric | 2871.2 ± 2.7 | 2871.2 ± 0.3 | 2874.8 ± 0.2 |
| $CH_{2,}$stretching, antisymmetric | 2921.2 ± 0.5 | 2916.7 ± 0.1 | 2919.8 ± 1.6 |
| $CH_{3,}$stretching, antisymmetric | 2957.2 ± 1.8 | 2957.0 ± 0.2 | 2959.0 ± 0.4 |

All wave numbers are in $cm^{-1}$.

## DISCUSSION

The region between 900–1185 $cm^{-1}$ was found to be of diagnostic importance in the case of cervical cancer and colon cancer[9,14,15]. However, in these tissues, the natural evolution of cells involves a change in levels of glycogen, carbohydrates, and glycoproteins as the cells mature and exfoliate. In the case of epidermal cells, such significant changes in levels of carbohydrates are not known. Thus, it is not surprising that carbohydrate levels would not be of diagnostic value and, in turn, the ratios based on these biochemicals as shown in Fig.1 would not be a very suitable biomarker. Changes in levels of phosphate during transformation of cell lines from normal to malignant states had been observed earlier[16,17]. Thus, the changes in the absorbance are significant between the nevus and melanoma as seen at 1080 $cm^{-1}$ absorbance intensity. This absorbance has been shown to be due to phosphates of nucleic acids[11,15] and identified as a useful parameter to distinguish between normal and cancer cells/tissues. Similarly, the ratio of RNA/DNA was found to be a suitable marker for distinction of normal and cancer[9]. In the present work, this ratio was also significantly different between the epidermis and the nevus/melanoma when the mean values were considered and the data analyzed by t test. The test showed that the mean value of the parameters were significantly different from each other. However, in case of medical conditions, patients can have small changes and still be positive for melanoma as seen from Figs. 3a–d. Thus, these parameters alone were not foolproof markers for differentiating the nevus from the melanoma though they were effective in distinguishing the normal from the abnormal as the changes in levels of these metabolites could vary between patients (see Figs. 1, 2, and 3). Therefore, it is important to identify additional parameters or features.

In the present work, we highlight on the monitoring of shifts of absorbance bands in case of diseases like compound nevus and melanoma, which not only have their location on the same organ, but are often difficult to diagnose. Moreover as seen from our observations, FTIR-MSP spectral features can be similar for such closely resembling diseases, making it further important to use as many parameters as possible for cross-verification to make the method more objective and potent in disease diagnosis.

Mcintosh et al.[7] had shown that the absorbance bands assigned to collagen (1035, 1080, 1204, 1240, 1280, and 1330 $cm^{-1}$) were only observed in the dermis. The absence of collagen from dermis, which could interfere with these ratios, was verified by the second derivative spectra that showed absence of peaks at 1204, 1280, and 1330 $cm^{-1}$. The sections from the biopsies studied in the present work were from the skin surfaces without involvement of the dermis. The FTIR-MSP further validates this point and acts as a cross-check to see whether the sections were from the surface or the deeper layers.

Image reassembling techniques have been successfully shown to form contrasting images of normal and malignant tissues of skin and cancer[18]. However, in our work, we hypothesized that such reassembling techniques would be greatly affected due to the similarity of the spectral features and methods like Artificial Neural Network (ANN), which will take into consideration not only the intensity, but also the shifts during the reassembly would be more suitable.

## ACKNOWLEDGMENTS

This research work was supported by the Israel Science Foundation (ISF Grant No:788/01) and the Israel Cancer Association (ICA).

## BIOSKETCHES

**Ziad Hammody, M.Sc.**, is a doctoral student investigating the occurrence of melanoma using optical systems such as FEWS and FTIR-MSP and has extensive experience in this field.

**Ranjit Kumar Sahu**, **Ph.D.**, is a postdoctoral research fellow in medical physics, working in the Department of Physics, Ben Gurion University of Negev, Beer Sheva, Israel. He is working on diagnosis of various forms of cancer using FTIR-MSP.

**Shaul Mordechai, Ph.D.**, is a full professor and heads the medical physics group in the Department of Physics and he is the Head of the Biophysics Center at the Faculty of Natural Sciences at Ben-Gurion University of the Negev, Beer-Sheva, Israel. His research interests are in the area of biomedical optics, FTIR-microscopy techniques, MID-IR spectroscopy, fluorescence spectroscopy, and medical informatics. E-mail: shaulm@bgu.ac.il. Website: www.bgu.ac.il/~shaulm.

**Emanuela Cagnano, M.D**., is a dermatopathologist and neuropathologist at the Department of Pathology, Soroka University Medical Center and is affiliated with the Faculty of Medicine, Ben-Gurion University of the Negev, Israel. Research interests are in dermatopathology and neuropathology. E-mail: emauelac@clalit.org.il

**Shmuel Argov, Ph.D.,** is the head of the laboratory at the Department of Pathology, Soroka University Medical Center and a lecturer at the Department of Health Sciences, Faculty of Medicine, Ben-Gurion University of the Negev, Israel. Research interests are in immunohistochemistry and cancer diagnosis and prognosis. E-mail: shargov@bgu.ac.il
